# Homeopathy for Depression: A Randomized, Partially Double-Blind, Placebo-Controlled, Four-Armed Study (DEP-HOM)

**DOI:** 10.1371/journal.pone.0074537

**Published:** 2013-09-23

**Authors:** Ubiratan C. Adler, Stephanie Krüger, Michael Teut, Rainer Lüdtke, Lena Schützler, Friederike Martins, Stefan N. Willich, Klaus Linde, Claudia M. Witt

**Affiliations:** 1 Institute for Social Medicine, Epidemiology and Health Economics, Charité – Universitätsmedizin Berlin, Berlin, Germany; 2 Teaching Hospital of the Charite – Universitätsmedizin Berlin, Head Center for Women’s Mental Health, Vivantes Humboldt Klinik, Berlin, Germany; 3 Karl and Veronica Carstens Foundation, Essen, Germany; 4 Institute of General Practice, Technische Universität München, Munich, Germany; 5 University of Maryland School of Medicine, Center for Integrative Medicine, Baltimore, Maryland, United States of America; University of Pennsylvania, United States of America

## Abstract

**Background:**

The specific clinical benefit of the homeopathic consultation and of homeopathic remedies in patients with depression has not yet been investigated.

**Aims:**

To investigate the 1) specific effect of individualized homeopathic Q-potencies compared to placebo and 2) the effect of an extensive homeopathic case taking (case history I) compared to a shorter, rather conventional one (case history II) in the treatment of acute major depression (moderate episode) after six weeks.

**Methods:**

A randomized, partially double-blind, placebo-controlled, four-armed trial using a 2×2 factorial design with a six-week study duration per patient was performed.

**Results:**

A total of 44 from 228 planned patients were randomized (2∶1∶2∶1 randomization: 16 homeopathic Q-potencies/case history I, 7 placebo/case history I, 14 homeopathic Q-potencies/case history II, 7 placebo/case history II). Because of recruitment problems, the study was terminated prior to full recruitment, and was underpowered for the preplanned confirmatory hypothesis testing. Exploratory data analyses showed heterogeneous and inconclusive results with large variance in the sample. The mean difference for the Hamilton-D after 6 weeks was 2.0 (95%CI −1.2;5.2) for Q-potencies vs. placebo and −3.1 (−5.9;−0.2) for case history I vs. case history II. Overall, no consistent or clinically relevant results across all outcomes between homeopathic Q-potencies versus placebo and homeopathic versus conventional case taking were observed. The frequency of adverse events was comparable for all groups.

**Conclusions:**

Although our results are inconclusive, given that recruitment into this trial was very difficult and we had to terminate early, we cannot recommend undertaking a further trial addressing this question in a similar setting.

Prof. Dr. Claudia Witt had full access to all the data in the study and takes responsibility for the integrity of the data and the accuracy of the data analysis.

**Trial registration:**

clinicaltrials.gov identifier NCT01178255.

***Protocol publication:*** http://www.trialsjournal.com/content/12/1/43

## Introduction

Depression is the most disabling mental disorder in the European Union (EU), affecting almost 7% of the EU population each year [1]. Homeopathy is sought by patients as complementary or alternative treatment for depressive disorders [2]–[9].

In classical homeopathy, the treatment consists of two main elements: taking case histories and prescribing individually selected homeopathic medicines [10]. The homeopathic case history aims to ascertain the totality of signs and symptoms of each patient, enabling the selection of an individualized homeopathic medicine. In addition, it attempts to understand the patients background, environment and daily routine. In some recent approaches the patient might complete a questionnaire prior to the medical consultation to improve the efficiency of obtaining the case history [11]. Homeopathic medicines are produced through sequential agitated dilutions in Decimal (D), Centesimal (C) or Quinquagintamillesimal (Q or LM) potencies.

Although there is no rationale for a mechanism of highly diluted homeopathic medicines, a relevant proportion of patients seeking a physician providing homeopathic care in Germany (6% of women and 5% of men) [12] and UK [2] suffer from depressive symptoms.

To date, only one full scale study, of which we are aware, on homeopathy for depression has been published [13]. The authors of this study concluded that individualized homeopathic Q-potencies are inferior to the antidepressant fluoxetine in a sample of patients with moderate to severe depression. This trial had no placebo control and interestingly, responder rates (defined as a decrease of at least 50% from baseline on the Montgomery & Åsberg depression rating scale) of both the homeopathic medicine and fluoxetine groups were higher (homeopathy 84.6%; fluoxetine 82.8%) than those usually found for antidepressants in trials (43–75%) [13], presuming a strong placebo effect. One might speculate that this could be due to the more extensive homeopathic case history in the study that compared homeopathic medicines with fluoxetine. However, whether individualized homeopathic Q-potencies and/or the type of the homeopathic case history have a specific therapeutic effect in acute depression is still unclear.

We investigated the 1) specific effect of individualized homeopathic Q-potencies compared to placebo and 2) the effects of an extensive homeopathic case taking (case history I) compared to a shorter, rather conventional one (case history II) in the acute treatment of major depression (moderate episode) after six weeks.

## Methods

The protocol for this trial and supporting CONSORT checklist are available as supporting information; see Checklist S1 and Protocol S1.

### Study Design

A randomized, partially double-blind, placebo-controlled, four-armed trial using a 2×2 factorial design with a six week study duration per patient was performed. Patients were randomized to one of four groups: 1) homeopathic Q-potencies/case history I, 2) placebo/case history I, 3) homeopathic Q-potencies/case history II and 4) placebo/case history II. The study protocol was published [14].

### Randomization and Blinding

A block randomization with variable block lengths was carried out using a 2∶1∶2∶1 ratio (exposing a smaller number of participants to placebo) and placed in sequentially numbered, sealed opaque envelopes. The randomization list was generated with SAS/BASE Software (SAS Inc., Cary NC, USA) by a statistician not further involved in the study.

The patients, the whole study team including the psychiatrist, the psychologist who assessed the HAM-D, and the statistician remained blinded to the identity of the four treatment groups until the end of the study. Only the study physician was unmasked for the case history type. The randomization list was kept strictly confidential. Only the study pharmacist and the statistician who generated the randomization list and prepared the envelopes had access to the randomization list. During the study, unblinding was allowed in the case of a patient emergency using sealed emergency envelopes, but this situation did not occur.

### Study Population

Eligible patients included men and women aged between 18 and 65 years diagnosed with major depression by a psychiatrist and rated afterwards as moderately severe (HAM-D 17 to 24) by a psychologist. Patients must not have been taking antidepressants or anxiolytic drugs (with the exception of Lorazepam as rescue medication, maximum dose 1.5 mg/day) at the time of inclusion. Capability and willingness to give informed consent and to comply with the study procedures were also required.

Exclusion criteria included schizophrenia or other psychotic disorders, bipolar affective disorder, schizoaffective disorders, alcohol or other substance abuse, eating disorders, a clinically significant DSM-Axis II (Diagnostic and Statistical Manual of Mental Disorders) disorder at the time of inclusion; severe depression which previously motivated a suicide attempt as defined by the Columbia-Suicide Severity Rating Scale (C-SSRS); suicidal ideation of type 4 or 5 in the C-SSRS [15], up to three months before screening; a clinically significant acute or chronic disease that would hinder regular participation in the study; treatment with antipsychotics, antidepressants, sedatives/hypnotics or mood stabilizers four weeks prior to the screening; complementary or alternative treatment used simultaneously to the study (for example acupuncture, phytotherapy, etc.); homeopathic treatment eight weeks prior to study entry; psychotherapy; simultaneous participation in another clinical trial (the last participation in a previous clinical trial must be completed at least three months prior to screening); concomitant pregnancy or breastfeeding; patients who are assumed to have a linguistic, intellectual or any other reason for not understanding the meaning of the clinical trial and for not complying with the necessary study procedures; persons who have been institutionalized by a court order; patients with an application for a pension. Recruitment strategies included close co-operation with outpatient practices, radio and television interviews on the topic, as well as advertisements in newspapers and underground trains.

### Regulatory and Ethical Approval

Written informed consent was obtained from all patients. Regulatory approval was received by the Bundesinstitut für Arzneimittel und Medizinprodukte (BfArM), EudraCT Nr: 2009-017458-11, Submission-Nr.: 4036175. Ethical approval was given by Ethics Committee, Berlin, Landesamt für Gesundheit und Soziales (LaGeSo): ZS EK 15 099/10. This study was in compliance with the Helsinki Declaration and with the ICH-GCP guidelines. Trial registration: clinicaltrials.gov identifier NCT01178255.

### Interventions

Patients were interviewed and treated at the Integrative Medicine outpatient clinic (CHAMP) of the Charité – Universitätsmedizin Berlin, by a medical doctor specialized in homeopathy with 20 years experience in classical homeopathy. The medical doctor was also experienced in case history and analysis under double blind conditions [13]. All patients completed a questionnaire before each consultation. The content and structure of the questionnaire used in DEP-HOM followed Hahnemanńs case history instructions [16], with some additional questions stressing the symptoms of a depressive episode.

#### Type of case taking

The particular approaches of each case history differed in the time used for the semi-standardized questionnaire and the onsite patient-doctor interaction. The homeopathic case history (case history I) was a more extensive conversation (60–90 minutes), in which the patient was asked to speak about different aspects in the questionnaire, including stressful live events, development and details of psychological and physical symptoms, and information which was discussed as needed. This was different in the shorter, more conventional case history (case history II). Case history II took around 30 minutes and the same questionnaire as in case history I was read in silence by the attending physician, who asked questions only to elucidate information that was unclear, resembling a rather conventional case taking, conducted by a general physician, when the patient already has a psychiatric diagnosis of depression. The follow-up differed in the time used to assess the remaining symptoms, 10 or 30 minutes, for the conventional or the homeopathic case history, respectively. In the latter case, interpersonal or ongoing stressful life events were also extensively assessed.

### Medications

The selection of the individualized remedies (case analyses) was carried out after the case history, in the absence of the patient, based on the latest clinical-pharmaceutical protocol developed by Hahnemann, which includes the standardized use of ascending Q-potencies 8 [16], [17].

Q-potencies were provided by Dr. Zinsser Arzneimittel, (Freudenstadt, Germany) and were manufactured according to the methodology described by Hahnemann [16]. The prescription of the individualized homeopathic Q-potency was sent to the Charité university clinic pharmacy, together with the patient’s randomization number. According to the randomization number, the study pharmacist dissolved one sucrose globule of the prescribed Q-potency (Q2) or one sucrose globule (placebo) in 10 ml of 20% alcohol-distilled water solvent. The vial was then labeled and sent to the study center, which was responsible for dispensing it to the patient within three days of the first case history.

The standard dose was one drop of the received vial three times per week [16]. Follow-ups were at two, four and six weeks after the first clinical interview. Blinded changes of medicine, potency and frequency with which the medicine should be taken were allowed on a clinical basis [16], [17].

### Outcomes

For the planned study, the primary endpoint was the mean total depression score using the 17-item version of the Hamilton Depression Rating Scale (HAM-D) [18] after six weeks. Severity of symptoms was assessed by a blinded investigator (psychologist) supervised by the Clinic for Psychiatry and Psychotherapy at Charité – Universitätsmedizin Berlin. The NICE (National Institute for Health and Care Excellence) threshold of 3 points was considered as parameter of clinical importance for the primary endpoint [19]. The secondary end points were the mean HAM-D total scores after two and four weeks, response (decrease of 50% or more from baseline HAM-D score) and remission (HAM-D scores ≤7) rates, Beck Depression Inventory (BDI) [20] total score and mean SF-12 Health Survey (SF-12) at weeks two, four and six. The minimum clinically important difference considered for SF-12 was 5 points [21].

Adverse events were collected during the study and will form part of the secondary endpoint data in determining the safety of homeopathic medicines. Participantś treatment expectations at baseline were also assessed.

### Statistics

For this study we assumed that the verum treatment is better than placebo by 2.7±6.0 (mean ± standard deviation) HAM-D score points after 6 weeks (corresponding to a SMD = 0.45), that type II case history is better than type I by 2.7±6.0 score points (SMD = 0.45), and that both effects do not interact. If so, a Bonferoni-adjusted F-Test (multiple significance level a = 0.05, two-sided) has a power of 83.5% to detect the difference between verum and placebo and a power of 85.0% to detect the difference in case history taking, if 68 patients are included in groups 1 and 3, and 34 patients are included in groups 2 and 4. This led to a total number of 228 patients, if one allows for a 10%drop-out rate per group. [14]. Because the preplanned sample size could not be reached and the study was underpowered for the preplanned primary confirmatory statistical hypothesis testing, the analyses plan was adapted accordingly and approved together with the earlier trial termination by the ethics committee and (ZSEK15099/10 June 24, 2010) and the Federal Institute for Drugs and Medical Devices (4036175 June 25, 2010). All data were analyzed solely descriptively without any formal hypothesis testing. Generalized linear models (GLM) with two factors were fitted to each continuously scaled outcome measure (HAM-D-Score, BDI-Score, SF-12-Score). Here the time point was modeled as a within-group factor, type of case taking and type of medication (verum, placebo) as between group factors, and the respective baseline value and the patients expectation as linear covariates. Normal distribution was assumed and Generalized Estimation Equations (GEE) were used to estimate differences between types of case taking and type of medication. For dichotomous outcomes (responder rate, remission rate) similar GLM were fitted, but the underlying distribution was assumed as binomial and the logit was taken as the link-function. Sensitivity analyses included a.) monotonic transformations of the outcome parameters (Box-Cox transformations), b.) adding various covariates to the model (sex, age, duration of disease, seasons when treatment took place, etc.), c.) changing the study populations (intention-to-treat, per-protocol, complete cases, etc.), and d.) use of alternative covariance matrices (autoregressive, uncorrelated). As this was an explanatory analysis, no p-values are reported. Thus, no multiple adjustments are needed.

## Results

**Figure 1:**
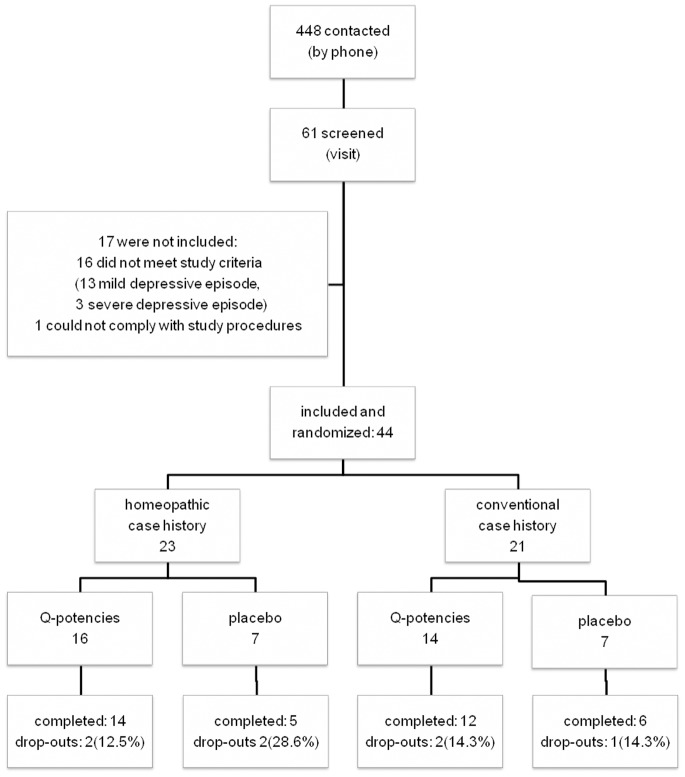
Flow diagram of subject progress through the trial.

Recruitment started in September 2010 and the study had to be terminated earlier than anticipated (March 2011), because it became clear that the planned recruitment strategy was not feasible. The rate of recruited patients per month was much lower than preplanned: less than 100 patients would have been included by February 2012, the already postponed end of the recruitment phase, as shown in Figure 1.

**Figure 2:**
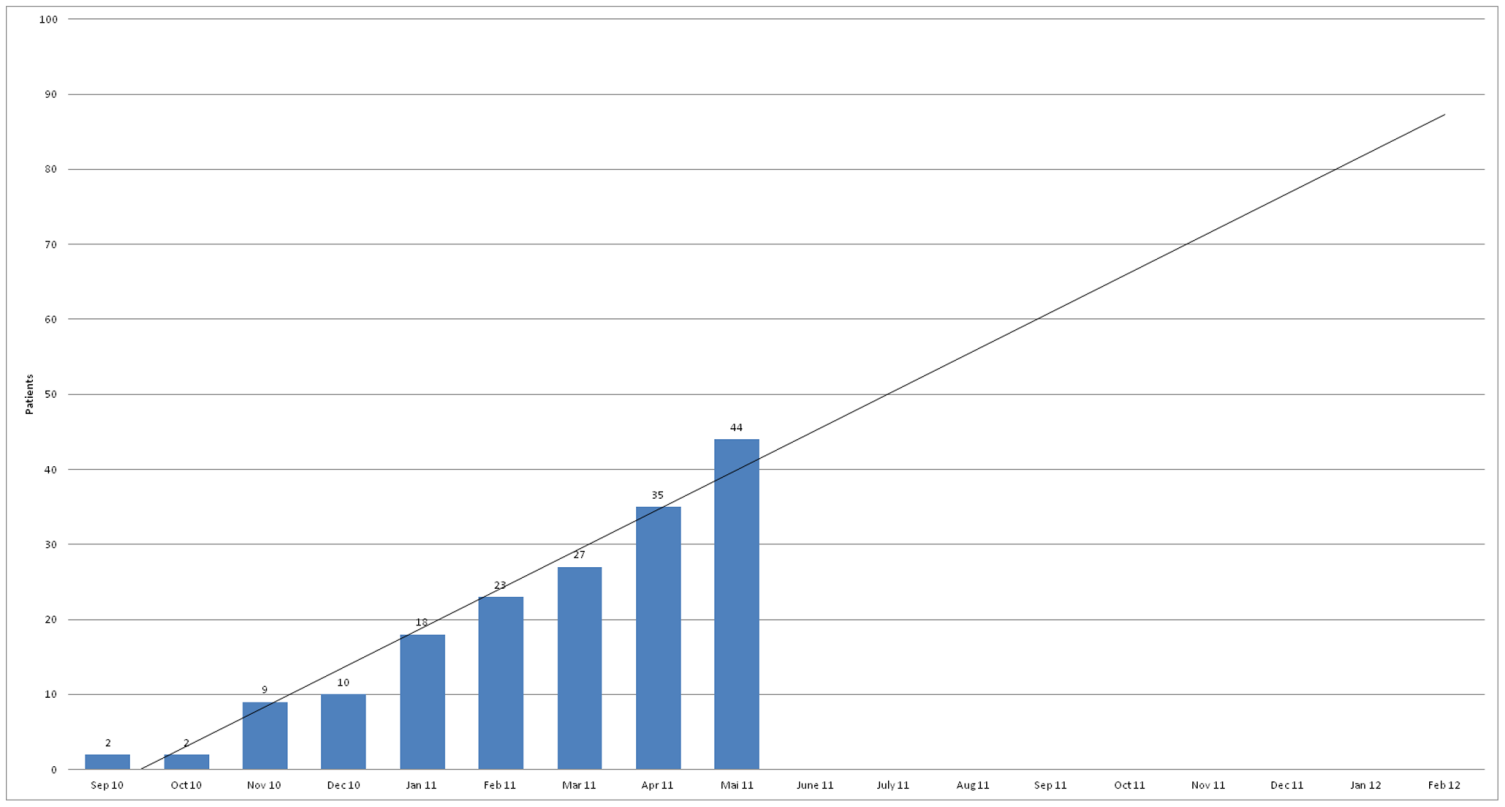
Trend line (cumulative) of included patients/month until February 2012 (end of the recruitment phase).

448 subjects contacted the coordinating study center and were screened by phone. 211 did not meet the eligibility criteria and 176 were not further interested after receiving more information about the study details. 61 patients participated in a screening visit and 44 were randomized. 17 were not included, because 16 did not meet study criteria (13 with mild depressive episode and 3 with a severe depressive episode) and 1 could not comply with study procedures. A total of 37 patients completed the 6-week treatment phase (Figure 2).

Included patients were mainly female (72.7%) and had an average age of 46.5 (SD 10.6) years. The average disease duration was 8.9 (SD 10.0) years. Further baseline data of study population are summarized in Table 1.

**Table 1:** Baseline demographics and clinical characteristics.

	Homeopathic case history		Conventional case history	
	+ Q-potencies	+ Placebo	+ Q-potencies	+ Placebo
Intention-to-treat population	n = 16	n = 7	n = 14	n = 7
Gender (n/% female)	13/81.3%	4/57.1%	11/78.6%	4/57.1%
Age (mean±sd)	49.6±9.2	45.4±11.2	43.1±11.5	47.3±11.1
Blood pressure (systolic) (mean±sd)	123.1±17.4	123.6±17.9	123.6±18.6	125.7±23.7
Blood pressure (diastolic) (mean±sd)	75.9±10.5	75.7±11.3	75.0±10.1	78.6±8.9
Other relevant diagnoses (n/%):	8/50.0%	1/14.3%	7/50.0%	4/57.1%
BMI (kg/m^2^)	24.6±7.1	23.8±1.9	24.6±4.5	25.0±5.1
Partnership: yes/%	7/43.8%	4/57.1%	5/35.7%	4/57.1%
Currently employed: yes/%	12/75.0%	5/71.4%	6/42.9%	5/71.4%
Duration of depression (years, mean±sd)	4.5±4.8	6.0±4.8	11.5±9.5	16.8±16.8
HAM-D (total score) (mean±sd)	19.9±2.8	19.0±1.4	19.4±2.3	19.0±1.8
BDI (total score) (mean±sd)	28.8±8.8	28.4±6.6	29.5±6.4	26.4±8.2
SF-12 psychic score (mean±sd)	35.2±5.1	30.9±4.2	33.7±6.3	33.7±7.4
SF-12 physical score (mean±sd)	34.4±9.0	45.6±7.9	38.7±8.8	44.3±11.0
Reasons for participation (n/%):				
curiosity	5/31.3%	4/57.1%	7/50%	3/42.9%
contribution to science	3/18.8%	2/28.6%	7/50%	4/57.1%
free therapy	5/31.3%	3/42.9%	2/14.3%	2/28.6%
improvement of symptoms	16/100%	5/71.4%	14/100%	4/57.1%
Appraisal of the effectiveness of homeopathy (n/%):				
very effective	3/18.8%	1/14.3%	1/7.1%	1/14.3%
effective	13/81.3%	4/57.1%	11/78.6%	2/28.6%
less effective	0/0.0%	1/14.3%	2/14.3%	3/42.9%
ineffective	0/0.0%	1/14.3%	0/0.0%	0/0.0%
Expectations (n/%):				
cure	0/0%	0/0.0%	1/7.1%	0/0.0%
marked improvement	11/68.8	4/57.1%	11/78.6%	2/28.6%
light improvement	5/31.3%	2/28.6%	2/14.3%	4/57.1%
no improvement	0/0.0%	1/14.3%	0/0.0%	1/14.3%

The following remedies were chosen by the physician: Alumina, Anacardium orientale, Aurum foliatum, Baryta carbonica, Calcarea carbonica, Carbo animalis, Colocynthis, Graphites, Kalium carbonicum, Lycopodium clavatum, Natrum carbonicum, Natrum muriaticum, Nitri acidum, Nux vomica, Phosphorus, Platina, Pulsatilla pratensis, Sepia succus, Silicea terra and Sulphur.

### Efficacy Measures

**Figure 3:**
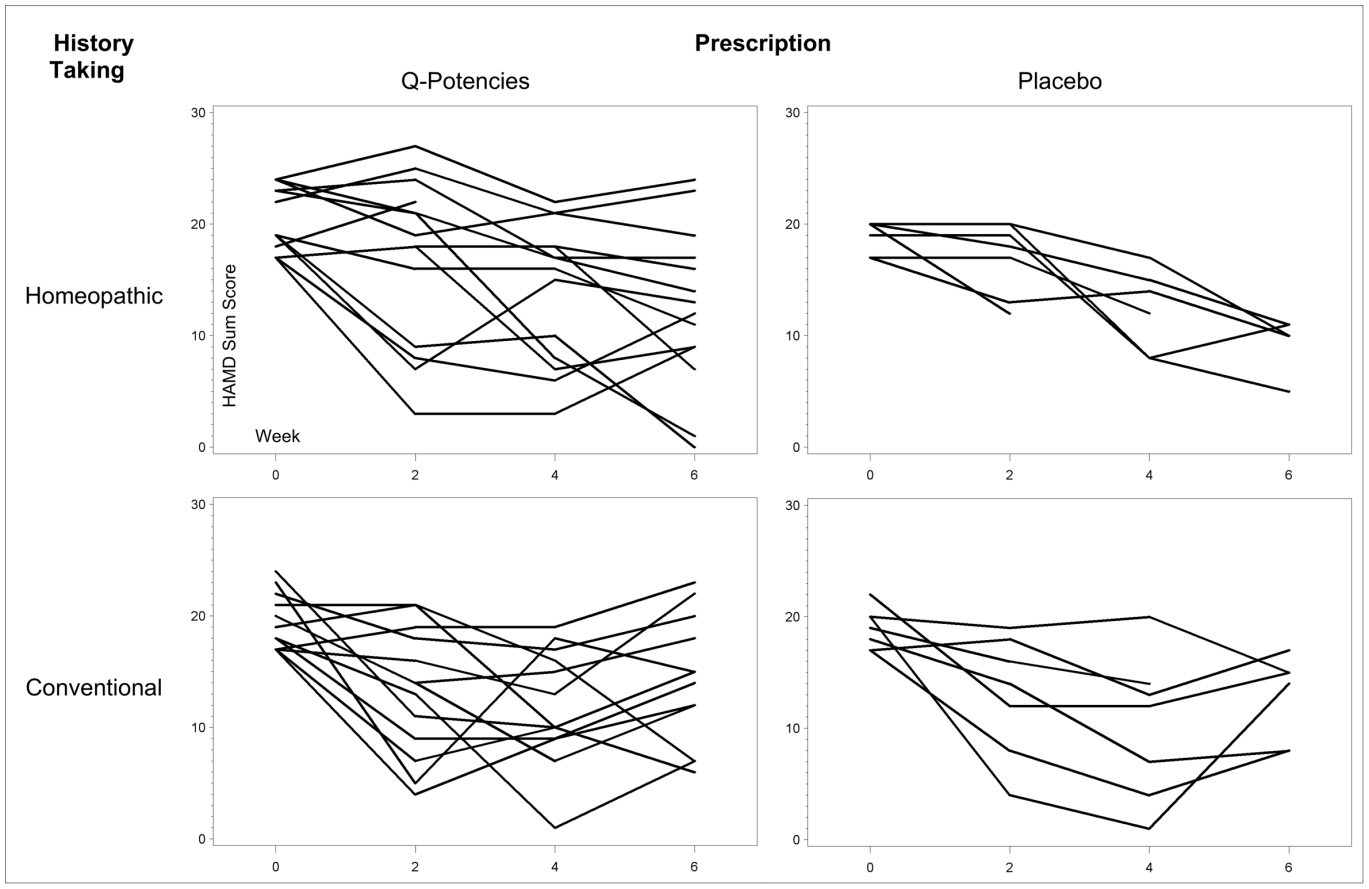
HAM-D development (x axis) over time (y axis) baseline and weeks 2, 4, 6 in the four groups for the single cases.

Results are exploratory and were heterogeneous with large variance in the sample (see Table 2 for raw data from continuous measures and Figure 3 for the HAM-D development over time in the single cases).

**Table 2:** Unadjusted outcomes after 2, 4 and 6 weeks for all four groups.

Measure	Q-potencies+homeopathic case history (mean ± sd)	Placebo+homeopathic case history (mean ± sd)	Q-potencies+conventional case history (mean ± sd)	Placebo+conventional case history (mean ± sd)
HAM-D week 2	17.1±7.1	17.0±3.2	13.2±5.7	13.0±5.4
HAM-D week 4	13.7±6.1	12.3±3.7	11.8±5.0	10.1±6.5
HAM-D week 6	12.5±7.1	9.4±2.5	14.3±5.7	12.8±3.8
BDI week 2	22.9±11.5	23.7±5.1	18.3±9.6	14.1±9.9
BDI week 4	18.1±12.0	17.0±10.8	16.0±8.8	12.3±9.9
BDI week 6	16.1±12.7	10.6±6.7	14.2±10.5	17.5±11.7
SF-12 mental summary scoreweek 2	36.6±9.7	32.6±8.6	42.0±8.6	39.7±7.3
SF-12 mental summary scoreweek 4	40.2±12.4	42.6±10.7	40.1±10.7	38.6±6.9
SF-12 mental summary scoreweek 6	41.8±11.0	46.1±10.6	41.0±13.6	39.6±11.6
SF-12 physical summary scoreweek 2	41.8±9.5	42.4±11.2	39.1±7.8	42.8±11.8
SF-12 physical summary scoreweek 4	44.7±10.6	43.6±9.2	42.7±9.0	39.1±13.3
SF-12 physical summary scoreweek 6	42.8±11.2	50.1±6.6	45.9±9.0	46.3±12.1

When calculating differences between groups, no relevant differences were observed between homeopathic medicines and placebo regarding HAM-D (estimated mean scores after 6 weeks 2.0 (95%CI −1.2;5.2) or BDI (after 6 weeks: 0.2 (−5.8;6.2, Table 3). Odds rations for response and remission rates, as well as most SF-12 results seemed to be slightly better in the homeopathic Q-potencies group, but taking into account the large confidence intervals and the descriptive way of data analyses (Table 3). This is similar for the comparison of both case histories where is seemed that the more conventional approach (case history II) is more beneficial (Table 3). Response and remission rates are shown in Table 4.

**Table 3:** Outcomes after 2, 4 and 6 weeks: mean differences and confidence intervals of pooled groups (HAM-D = Hamilton Depression Scale values <0 favor homeopathy or case history I, BDI = Beck Depression Inventory values <0 favor homeopathy or case history I, SF-12 = short form 12 health related quality of life questionnaire values >0 favor homeopathy or case history I).

	Homeopathic Q-potencies vs placebo	Homeopathic (case history I) vs conventional case history (case history II)
Measure	Mean differences (95% CI-limits)	
HAM-D week 2	−0.1 (−3.5; 3.3)	3.7 (0.7; 6.8)
HAM-D week 4	1.8 (−1.5; 5.2)	1.9 (−1.3; 5.2)
HAM-D week 6	2.0 (−1.2; 5.2)	−3.1 (−5.9; −0.2)
HAM-D weeks 2–6	1.3 (−1.5; 4.0)	0.9 (−1.5; 3.3)
BDI week 2	0.6 (−3.8; 5.0)	6.3 (1.9; 10.8)
BDI week 4	1.7 (−4.6; 8.0)	2.8 (−3.6; 9.3)
BDI week 6	0.2 (−5.8; 6.2)	−2.2 (−8.4; 4.0)
BDI weeks 2–6	0.8 (−4.1; 5.7)	2.3 (−2.8; 7.4)
SF-12 mental summary score week 2	2.7 (−2.7; 8.2)	−6.0 (−11.3; −0.7)
SF-12 mental summary score week 4	−0.8 (−6.7; 5.0)	2.3 (−3.8; 8.4)
SF-12 mental summary score week 6	−1.8 (−8.6; 4.9)	3.9 (−2.4; 10.2)
SF-12 mental summary score weeks 2–6	0.0 (−4.7; 4.7)	0.1 (−4.5∶4.6)
SF-12 physical summary score week 2	2.8 (−3.1; 8.7)	1.9 (−3.7; 7.6)
SF-12 physical summary score week 4	7.3 (2.4; 12.2)	4.0 (−1.1; 9.2)
SF-12 physical summary score week 6	1.1 (−3.7; 5.9)	1.1 (−3.8; 6.0)
SF-12 physical summary score weeks 2–6	3.7 (−0.2; 7.6)	2.4 (−1.6; 6.3)
Response rates (HAM-D reduction >50%)	**Odds Ratio (95% CI-limits)**	
Response week 2	4.47 (1.18; 16.90)	0.24 (0.08; 0.69)
Response week 4	1.08 (0.24; 4.80)	0.64 (0.17; 2.46)
Response week 6	0.88 (0.17; 4.58)	0.81 (0.17; 3.81)
Response week 2–6	1.62 (0.51; 5.18)	0.50 (0.20; 1.28)
Remission rates (HAM-D <8)	**Odds Ratio (95% CI-limits)**	
Remission week 2	2.64 (0.77; 9.02)	0.35 (0.11; 1.08)
Remission week 4	1.49 (0.46, 4.84)	0.28 (0.09; 0.85)
Remission week 6	3.95 (0.90; 17.45)	2.27 (0.49; 10.57)
Remission week 2–6	2.49 (1.11; 5.58)	0.60 (0.28; 1.29)

**Table 4:** Individualized homeopathic medicines vs. placebo/type of case history: response and remission.

	Q.potencies vs placebo/homeopathic case history	Q.potencies vs placebo/conventional case history
Measure	Odds Ratio (95% CI-limits)	
Response week 2	10.45 (3.48; 31.33)	1.92 (0.22; 17.08)
Response week 4	1.07 (0.14; 8.18)	1.09 (0.14; 8.13)
Response week 6	0.76 (0.08; 7.15)	1.03 (0.10; 10.50)
Remission week 2	4.45 (1.38; 14.39)	1.56 (0.20; 12.23)
Remission week 4	7.76 (2.14; 28.15)	0.28 (0.04; 1.80)
Remission week 6	1.40 (0.09; 22.37)	11.18 (3.13; 39.97)

### Safety

The number of patients reporting an Adverse Event (AE) was similar in the homeopathic medicine groups (19/30 = 63.3%) and the placebo groups (9/14 = 64.3%). A mean of 1.23 AE/patient (37/30) was documented in the homeopathic medicine groups and 1.07 (15/14) in the placebo groups. No serious AE (SAE) was reported or observed, nor did any patient report suicide ideation during the study. Reported AE were acne (2×), herpes simplex, eczema (2×), psoriasis (worsening), hyperhidrosis, xerosis, polymorphous light eruption, hypertrichosis, erythema, skin hyperpigmentation, influenza, acute nasopharyngitis (2×), acute laryngitis, otalgia, anxiety, somatoform pain, somatoform autonomic dysfunction, irritability, nightmares, worsening of the depressive episode, exhaustion, narrowing of attention, polyphagia, pseudocyesis, unspecified abdominal pain, acute cystitis, hemorrhoids, tachycardia, cramps, headache, acute gastroenteritis (3×), superficial injuries, rupture of ligaments at ankle and foot level, anterior chest-wall pain, pain in limb, pain localized to upper abdomen, nausea (2×), and ocular pain.

## Discussion

This study was planned for confirmatory statistical hypothesis testing to determine whether homeopathic Q-potencies and/or the type of the homeopathic case history would have a specific effect in the treatment of acute major depression, but because the preplanned sample size was not reached and following a lack of recruitment the trial was underpowered for the primary statistical analyses, data could only be analyzed using exploratory statistical methods.

We are not aware of a prior trial that aimed to evaluate both the specific effect of homeopathic medicines and of a homeopathic case taking in patients with major depression. Advantages were a study protocol that is in accordance with the EMEA (European Medicines Agency) guidelines, which recommends placebo-controlled studies and the duration of six weeks for trials investigating medicines for depression [22]. Patients, study personnel and the rater for the primary outcome were blinded for both (type of medicine and case history); only the study physician was unblinded for the type of case history. Although performed in a highly controlled setting, this study allowed an individualized treatment approach with Q-potencies following the classical homeopathic approach.

Furthermore, patient safety played an important role in this study and was reflected by visits every 2 weeks and by the fact that depression severity was limited to a maximum HAM-D score of 24. For more severe depression the benefit of antidepressant over placebo is substantial [23]. People with moderate depression can also benefit from psychotherapeutic interventions, specifically cognitive behavioral therapy. In addition, structured group physical activity programs have also been shown to provide some relief from depressive symptoms [24]. In our sample, patients were referred to psychotherapy at the end of the study, if necessary.

Overall, depression seems to be a disease that has high placebo response and the difference between placebo and active treatment seems to be difficult to detect [25]. In our study the variance between the patients was large. Wide confidence intervals made it impossible to judge the exploratory results. Furthermore, in a small sample, results are more influenced by random variation. A clearer picture might have been observed in a much larger sample.

Interestingly, the results of the homeopathic case taking were less positive than expected. We would have expected a more positive outcome, because according to a systematic review [26], most studies evaluating the effect of patient-physician communication demonstrated a correlation between effective physician-patient communication and improved patient health outcomes. However, one other study has shown that psychosocial questions from the physician can be associated with a worsening of depression symptoms immediately following the interview [27]. Therefore, we can only speculate that the extensive homeopathic case history (and its higher volume of psychosocial communication) may have contributed to an increase in the depressed affect from pre- to post-case history at weeks 2 and 4, inasmuch as at these endpoints the psychological evaluation occurred immediately following the medical interview. At week 6, patients were first interviewed by the psychologist, who rated the depression scores, and then by the physician (end of study interview) and the homeopathic case taking showed better depression scores than the more conventional approach group. These results should be interpreted with caution because of the small sample and the large variance in the results.

The small sample is the most important limitation of our study. The study was planned based on the experience of other studies and the evidence that patients seeking a homeopathic physician frequently suffer from depression [2], [5], [12]. When planning the trial we were aware that recruitment for a placebo-controlled trial on depression could be difficult. Quality assurance of inclusion criteria required that patients were diagnosed by a psychiatrist; however, this requirement complicated the process and increased waiting time for patients who were seeking treatment. Furthermore, limiting the sample to moderate depression and having a long list of exclusion criteria had a negative impact on the recruitment, as depression in general is associated with significant medical and psychiatric comorbidity. Because of this we implemented an extensive recruitment strategy (incl. close co-operation with outpatient practices, radio and television interviews on the topic, as well as advertisements in newspapers and underground trains). However, it was not anticipated that two other clinical studies on depression also initiated recruitment in Berlin at the same time. Those industry-sponsored studies offered financial compensation to their subjects, a practice we decided to avoid because of its impact on selection bias.

With an inclusion/screening rate of 44/448 = 9.8%, far below rates usually observed in conventional studies on depression, (e.g. Zajecka et al. [28] had 511/1184 = 43%), this study highlights recruitment problems in placebo-controlled trials on homeopathy, also seen to be the case in other homeopathy trials. For example, in a placebo-controlled trial on classical homeopathy for patients with atopic dermatitis, Siebenwirth et al. were able to include only 24 of 746 screened patients because of narrow eligibility criteria [29]. In a trial on depression in the general practice setting in UK, Katz et al. expected over 230 suitable patients during the recruitment phase, but only 31 patients were referred for possible inclusion in the trial. Twenty-three met the entry criteria, 11 were randomized and 6 completed the study [30]. The authors concluded that a trial of this design in general practice is not feasible, because of recruitment difficulties, many of them linked to patient preference. Different approaches are required to recruit adequate patient numbers to trials of this sort.

Being fully aware that recruitment could be difficult when we began our study, we developed a systematic stepwise recruitment strategy that included referrals from colleagues with outpatient practices as well as extensive media presence (e.g. advertisements in the underground and in regional newspapers, and participation in regional television shows on health topics). However, the size of the recruited sample reveals that this recruitment strategy was also not successful.

## Conclusions

Although our results are inconclusive, given that recruitment for this trial was very difficult and necessitated early termination of the study, we cannot recommend undertaking a further trial addressing this question in a similar setting.

## Supporting Information

Checklist S1 **Consort Checklist.**
(DOC)

Protocol S1 **Trial Protocol.**
(PDF)
